# Proposal and Rationale for a Cardioethics Curriculum

**DOI:** 10.1016/j.jacadv.2024.100845

**Published:** 2024-02-02

**Authors:** Sarah C. Hull, J. Brendan Mullen, James N. Kirkpatrick

**Affiliations:** a Section of Cardiovascular Medicine, Yale School of Medicine, New Haven, Connecticut, USA; b Program for Biomedical Ethics, Yale School of Medicine, New Haven, Connecticut, USA; c American College of Cardiology, Washington, District of Columbia, USA; d Division of Cardiology, University of Washington, Seattle, Washington, USA; e Department of Bioethics and Humanities, University of Washington, Seattle, Washington, USA

**Keywords:** cardioethics, education, ethics

## Abstract

The modern practice of cardiovascular medicine involves many ethical controversies in the care of our complex patients. Accordingly, we propose a framework for a practical, clinically based “cardioethics” curriculum that might be incorporated into fellowship training to prepare cardiologists to cope with increasingly complex ethical dilemmas. This work can also be adopted into continuing medical education for cardiologists and other cardiovascular practitioners given the critical importance of collaborative care in cardiology. We discuss heart transplant allocation, futility concerns, withdrawing care, advance care planning, conflicts of interests, and distributive justice. Sound ethical decision-making in cardiology requires a combination of extensive technical knowledge, nuanced appreciation of individual patient goals and values, and thoughtful application of ethical principles and reasoning. Cardiologists have an exceptionally broad toolkit of medications and interventions to address high-stakes disease states. We should maintain a similarly broad ethical toolkit to provide the best care for our patients.

## Highlights

• Advances in cardiology have allowed us to provide ever greater care to ever more complex patients.
• These advances combined with the high-stakes nature of cardiac disease can lead to complex ethical dilemmas in cardiovascular practice.
• Cardiologists should understand the ethical principles and engage in the ethical reasoning involved in resource allocation, end-of-life care, conflicts of interests, and balancing autonomy with beneficence and justice.
• Complex ethical decision-making in cardiology requires technical knowledge, appreciation of patient goals and values, and thoughtful application of ethical principles.

Major technical advances focus cardiovascular discourse on what *can* be done for patients, often obscuring the equally important question of what *should* be done. While complex bioethical issues have been extensively addressed in critical care literature and practice, cardiology has just begun to scratch the surface. The 2020 American Heart Association and American College of Cardiology Consensus Conference on Professionalism and Ethics brought welcome attention to the importance of ethics in the practice of cardiology with a focus on contemporary issues such as diversity, equity, and inclusion.^1^ Here, we propose a framework for a “cardioethics” curriculum that might be incorporated into fellowship training to prepare cardiologists to cope with increasingly complex ethical dilemmas that they are likely to face in practice.

To be clear, ethical dilemmas often emerge when there are seemingly reasonable arguments supporting divergent courses of action or opposing moral claims. But it would be a mistake to conclude that there are therefore “no right answers” in succumbing to the fallacy of false equivalences and moral relativism. Indeed, even though a perfect solution may not always be achievable, there are clearly better and worse approaches to addressing many ethical dilemmas as we will explore in the following sections. While there are no perfect therapies and few cures in cardiovascular medicine, applying the best available evidence and sound clinical reasoning allows us to markedly improve clinical outcomes. Similarly, the application of thoughtful moral reasoning through the acquisition of practical ethical skills has the potential to significantly improve ethical outcomes as well, in addition to preserving the moral integrity of both clinicians and patients.

Accordingly, we propose that cardioethics content be incorporated more consistently and intentionally within cardiology education at multiple levels. This would ideally include at least quarterly conferences for cardiology fellows as part of their formal didactic curriculum during training, as well as dedicated programming at conferences and scientific sessions for continuing medical education. Recognizing that cardiology faculty and trainees may come from varying backgrounds with respect to ethics education, we have prepared practical primers on core principles in moral philosophy and bioethics (Tables 1 and 2) to underscore their relevance to current clinical ethics. Understanding the origin of these principles, as well as their respective advantages and pitfalls, can help to equip faculty and fellows alike to engage in more deliberate moral reasoning rather than relying on emotion or instinct. We also provide potential cases with discussion guides as a starting point for curricular development (Table 3), which could be integrated with additional content such as journal clubs, fellow case presentations, and discussion of current events affecting the delivery of health care. While certainly not an exhaustive list, we suggest that a reasonably comprehensive cardioethics curriculum address at least the following general topics in professional, clinical, and systemic medical ethics: heart transplant allocation, futility concerns, withdrawing life-sustaining treatments, advance care planning, conflicts of interest regarding referrals and procedures, and distributive justice and fairness (Central Illustration). We focus on these domains because they are of particular salience in clinical cardiology as we will endeavor to illustrate with relevant examples in the descriptive sections to follow, with corresponding didactic cases in Table 3. While this proposal is primarily focused on curricular content, we have described our rationale in further detail and proposed more specific pedagogical approaches in a recent commentary in this journal.^3^

**Table 1:** Moral Philosophy Primer

Philosophical Approach	Description
*Aristotelian or virtue ethics*	Aristotle postulated that moral behavior should be guided by virtues or positive character traits to promote human flourishing (“eudaimonia”) and achieve one’s destiny or purpose (“telos”) as a virtuous moral agent. A virtue ethicist would justify his or her actions by aligning them with the comportment of a decidedly virtuous person who strives to do the right thing for the right reason because it is a core component of his or her character. Such virtues might include honesty, generosity, and courage. An ancient theory of great historical interest, it is less cited in modern moral philosophy. Nonetheless, virtue ethics are often implicit in discussions regarding professional values and defining character traits of the aspirational physician.
*Deontology*	A duty-based approach to moral philosophy most famously articulated by Immanuel Kant who proposed the “categorical imperative” as a framework for moral behavior. The first formulation of this imperative, “Act only according to that maxim whereby you can at the same time will that it should become a universal law” is similar to the so-called Golden Rule (“treat others how you would like to be treated”). Kant also proposed that rational humans, as autonomous moral agents, have a duty to treat other rational humans as ends in themselves rather than simply as means to end. This is a commonly cited underpinning of the concept of autonomy as “respect for persons.”
*Consequentialism and utilitarianism*	Championed by Jeremy Bentham and John Stuart Mill, consequentialist theory focuses generally on the outcomes of actions in determining their morality, and utilitarianism specifically emphasizes “maximizing” outcomes or acting in a way to provide the “greatest good for the greatest number.” While often a practical approach in public health ethics when applied on a population level, it can become problematic on an individual level when “the ends justify the means” and virtue ethicists or deontologists object to the means as unethical. Utilitarians might counter that deontologists may be insufficiently willing to “get their hands dirty” when it is necessary to make difficult choices or compromises for the sake of the greater good. However, it is also increasingly recognized that strict utilitarian approaches may further disadvantage individuals from medically underserved backgrounds who have worse outcomes due to social determinants of health.
*Contractarianism and theory of justice*	John Rawls was a political philosopher who built upon the Hobbesian concept of social contracts as well as Kantian ethics to conceptualize liberty as the freedom to act in a way that allows for self-determination without impinging upon the liberty of others to do so. His theory of justice as fairness is framed by the “veil of ignorance,” a thought experiment proposing that individuals should construct societal organization as if they had no prior knowledge of what position they might occupy in that society. He argued that individuals operating under a veil of ignorance would structure a just society in which privilege resulting from accidents of birth would not provide undue advantages and rather that all members of society would enjoy equality of opportunity.
*Case-based ethics*	This approach focuses on individual stories (“narrative ethics”) and their unique ethical considerations, often incorporating reasoning from prior cases (“casuistry”), rather than looking to overarching theories to provide moral guidance. This can be thought of as a “grassroots” approach rather than a “top-down” approach, and one that focuses on context and precedent over abstract theory.
*Feminist ethics*	An approach to moral philosophy that de-emphasizes traditionally “masculine” values such as rules, rights, and autonomy; instead placing greater value on interdependence, relationships, and responsibilities as well as amplifying traditionally marginalized female perspectives and voices.
*Principlism*	An applied approach to moral philosophy proposed by Beauchamp and Childress in the late 1970s, based on the Belmont Report for protection of human research subjects in response to the infamous Tuskegee study that ended earlier that decade. The aim of principlism was to distill core tenets of moral philosophy, notably duty-based or deontological theory and outcomes-based or consequentialist theory, into 4 prima facie principles of “common” morality including autonomy, beneficence, nonmaleficence, and justice. This has since become the dominant theory in modern medical ethics. While the 4 principles were originally intended to carry equal weight, autonomy has emerged in practice as the dominant principle in Western ethics. These principles are described in greater detail in Table 2.

**Table 2:** Principles of Medical Ethics

Ethical Principle	Description	Examples
*Autonomy or respect for persons*	The principle that every person is a moral agent of infinite value with the right to self-determination. This principle occupies a position of primacy in current Western medical ethics and is operationalized by the ubiquity of informed consent. Clinicians may not unilaterally impose or withdraw medically indicated treatment without the consent of a patient or surrogate.	A patient with refractory angina may elect to continue a conservative strategy with medical management alone even if her cardiologist strongly believes that percutaneous coronary intervention would be a better option.
*Beneficence and nonmaleficence*	The principles that clinicians have a duty to promote the health and wellness of their patients, and they also have a duty to avoid causing harm. Given that all treatments pose some theoretical risk of harm, implicit in this definition is the imperative to avoid net harm. Recognizing autonomy as a first principle, clinicians may not impose any treatment without a patient’s informed consent. However, when patients make a decision that appears to go against their best interests—when putative autonomy threatens to undermine beneficence—clinicians have a duty to interrogate whether that decision is truly informed.	An otherwise healthy middle-aged man presenting with an ST-segment elevation myocardial infarction who refuses coronary angiography due to fear of needles may not be weighing risks and benefits rationally with respect to his underlying goals. While this procedure should not be forced upon him, it would be irresponsible to accept his initial refusal at face value without ensuring that he fully understands the grave repercussions. Autonomy by definition requires that decision-making be fully informed.
*Justice and fairness*	The principle that limited resources should be stewarded and allocated both judiciously, so as not to waste them, and fairly, so as not to give undue advantages to certain members of society. In other words, patients should have equitable access to resources regardless of age, sex or gender identity, race or ethnicity, socioeconomic status, religion, and similar individual factors.	Addressing social determinants of health that perpetuate disparities in cardiovascular disease prevalence and outcomes, such as improving universal access to healthy food and education, is essential to achieve a more just distribution of resources.

**Table 3:** Examples of Cardioethics Cases

Case Scenario	Questions	Discussion
You are a heart failure specialist who has been caring for a 44-y-old man with progressive heart failure due to idiopathic dilated cardiomyopathy for several years. He now has symptoms at rest despite optimal medical management and cardiac resynchronization therapy. You think he would be an excellent candidate for heart transplant and advocate for listing him at your institution’s transplant meeting, but the transplant surgeon objects because the patient is a Jehovah’s Witness and will refuse blood transfusions. She cannot agree to proceed with transplant surgery in this patient if she will not be able to administer even a life-saving transfusion intraoperatively, as she is concerned about potentially harming this patient and potentially “wasting” an organ if he dies due to untreated blood loss intraoperatively.	Are the transplant surgeon’s concerns valid? Why or why not?	*The surgeon is voicing concerns based on the principles of nonmaleficence and justice. She could cause undue harm to a patient undergoing surgery with substantial bleeding risk if she is unable to treat blood loss with all possible measures including transfusion. Listing this patient for heart transplant may result in a heart being allocated away from another patient at lower perioperative risk based on willingness to accept transfusion, which in might make the other potential patient a better steward of this scarce resource. Conversely, one might argue that this reasoning is a form of religious discrimination, which also violates the principle of justice as fairness with equitable access to resources.*
	How might you and the transplant team be able to reconcile conflicting ethical considerations in this case?	*Clear and respectful communication, as always, is critical in this case. It is important for the transplant team members to express their concerns to this patient with an emphasis on harm reduction. It is equally important for the team to understand this individual patient’s preferences and values, since the degree to which different individuals practice the same religion can vary significantly. Does this patient truly object to blood transfusion under any circumstances or would he make an exception for a transplant? Would he be willing and able to bank his own blood in advance as an acceptable alternative? Avoiding broad assumptions and exploring alternative possibilities can often help to reconcile ethical dilemmas.*
You have been asked to consult on a 56-y-old woman with a history of intravenous drug use complicated by endocarditis requiring bioprosthetic mitral valve replacement a year ago. She was admitted yesterday with fever and dyspnea with blood cultures growing *Staphylococcus aureus*. Transesophageal echocardiogram revealed 2.5 cm vegetation on her mitral bioprosthesis with evidence of leaflet perforation and severe regurgitation. You therefore consulted cardiothoracic surgery, but the surgeon declined to operate, stating “this would be a futile operation because she’s just going to keep using drugs, and she will be back soon enough with the same problem.”	Do you agree with the surgeon? Why or why not?	*While it is certainly true that substance addiction is a chronic disease that significantly increases one’s risk for recurrent infective endocarditis (IE), this information is insufficient to conclude that valve replacement would be futile. It does not meet the standard for physiologic futility, as valve replacement (along with antibiotics) would indeed be very effective at treating acute IE. Determining nonphysiologic futility requires understanding underlying medical and psychosocial issues that might compromise longer-term efficacy, as well as understanding patient goals and values. For example, we do not know if this patient has ever been offered addiction treatment to prevent relapse, which is a critical component of the treatment of injection drug use-associated IE (IDU-IE). If she is willing to undergo addiction treatment, she may be able to reduce her likelihood of relapse by as much as 50%.**^2^*
	What are your ethical obligations toward this patient?	*Assuming she affirms the wish to be treated for her disease, it is our obligation to do so according to evidence and guidelines our profession has outlined as the standard of care. This includes both treatment of her acute problem, infective endocarditis (IE), as well as the underlying cause of this problem, injection drug use due to substance addiction. The updated 2020 ACC/AHA guidelines on the management of valvular disease state: “In patients with recurrent endocarditis and continued intravenous drug use, consultation with addiction medicine is recommended to discuss the long-term prognosis for the patient’s refraining from actions that risk reinfection before repeat surgical intervention is considered.*”*^2^**Furthermore, it should be noted that addiction medicine specialists can offer more than prognostication, as evidence-based interventions exist for the treatment of addiction. Until a patient has been offered multiple addiction treatments and has demonstrated consistent lack of response, it may be difficult to predict likelihood of relapse. Given that ACC/AHA guidelines also recommend early surgery for IE leading to valvular dysfunction with symptoms of heart failure, as is the case in this patient, both valve replacement surgery and addiction treatment should be offered to her during this admission.*
You have just admitted an 81-y-old man with end-stage heart failure due to transthyretin amyloidosis to the cardiac intensive care unit. This is his fourth admission in the past 6 mo, but he recently started tafamadis as his insurance finally approved it—he could not afford to take it otherwise. He has already been referred to advanced heart failure to consider mechanical support or transplant evaluation, but he was deemed to be a poor candidate for both due to advanced age, frailty, and multiple medical comorbidities. While he has told his family many times over the years that he would never want to “be a vegetable,” he has expressed to them recently that he would want “everything done” if he were to become critically ill because he is very hopeful about his prognosis now that he is on tafamidis (despite the guarded prognosis given by his outpatient cardiologist). He is lethargic with hypotension and signs of poor perfusion on exam consistent with cardiogenic shock, and he is not able to express his wishes or goals of care to you. In your opinion, he is unlikely to survive this hospitalization.	How should you proceed?	*When patients are unable to express their wishes or preferences, we use the “substituted judgment” standard by interrogating either prior documentation of wishes (such as a living will or advanced directives) or prior conversations with surrogate decision-makers. Based on the family’s description of the patient’s wishes, it may be reasonable to undertake a trial of aggressive medical therapy including inotropic support for a few days. However, physicians have a duty to be honest about the low likelihood of success to set reasonable expectations. Sometimes it can be helpful to offer a few days of full support to clarify prognosis (“let the dust settle” during a “time-limited trial”) and to allow other family members to make travel arrangements to be there in person. Ideally, outpatient clinicians would have asked the patient to define what he meant by “everything” and further explored his core values pertaining to longevity, in addition to his optimism concerning tafamidis. Though this was not done previously, it can be done with the surrogates now, to some extent.*
	After several days of inotropic support, afterload reduction, and diuresis titrated with a pulmonary artery catheter, the patient remains critically ill and shows no signs of improvement. You arrange a family meeting to address goals of care. How might you approach this discussion?	*If patients do not “rally” or show signs of improvement within 48 to 72 h, physicians can explain with more certainty that they are unlikely to recover despite reasonably aggressive medical therapy, and this may assuage feelings of guilt that family members may have for what they often describe as “letting go.” What may offer further relief is the reassurance that allowing patients to be comfortable and not asking them to “fight” any longer may be the most caring gesture families can make toward their loved one at the end of life. Finally, it is important to clarify that you are not just “withdrawing” care but rather shifting goals of care to maximize patient comfort and dignity, emphasizing our broad toolkit of palliative therapies such as opioids to treat air hunger.*
You are heading to clinic after a divisional meeting, during which the service chief mentioned that nuclear stress test volumes are down, and more business is needed to keep the nuclear cardiology program functional to provide high-level testing for patients with amyloid and infiltrative cardiomyopathies. Your first patient is transferring from another cardiologist to your outpatient general cardiology supervising attending. The patient has a history of coronary artery disease with a stent 3 y ago in the setting of chest pain and a nuclear perfusion stress test demonstrating a mild reversible defect (no history of myocardial infarction). The patient has had yearly nuclear perfusion stress tests thereafter demonstrating no ischemia. An echocardiogram performed last year was unremarkable. The patient is a highly placed executive at a well-known company and is referred by a boutique internal medicine physician who joins the appointment via teleconference. Initially perturbed that someone other than the senior cardiologist is involved in his care, the patient soon adopts a pleasant tone and answers your questions. There are no symptoms suggestive of angina and nothing in the medical history or physical examination to suggest significant asymptomatic progression of cardiovascular disease or elevated risk thereof. The internal medicine physician suggests you ask your preceptor to join the appointment so that a nuclear perfusion stress test can be ordered and the patient can return to work.	What ethical considerations come into play concerning the request for a nuclear perfusion stress test?	*The information provided in the question stem suggests that evaluation for coronary ischemia by stress testing would be rarely appropriate in this scenario. While the stent was placed >2 y prior, the patient had a negative stress test 1 y ago. There is a tendency to view frequent testing as better care, particularly if it can detect disease at an early stage. In this case, however, there are significant downsides, including cumulative exposure to radiation and the risk of false positive results, potentially leading to unnecessary invasive testing that carries small but significant risk. Furthermore, in the absence of symptoms, it is unclear what one would do with the results.* *Appropriate, patient-centered care is due to every patient, and patients who are socially advantaged should not be overtreated in a misguided attempt to placate them. This is a difficult situation, as we wish to treat all patients with respect and dignity, and entering into conflict may be unpleasant. This creates a conflict of interest with appropriate care, in addition to that represented by the financial needs of the nuclear lab. However, clinicians have a moral responsibility to uphold the principles of beneficence and nonmaleficence, and patient autonomy does not enshrine the right to demand inappropriate testing or treatment. While you may wish to consult your supervising physician before delving into a discussion about appropriate testing with the patient and internist, it will be necessary to address.*
You are approached by a pharmaceutical company to join a Speakers Bureau for a novel antihypertensive. The company will supply you with a professional slide set and arrange lectures in the community, but you are free to say what you wish while using the slide set. The company will pay you a handsome honorarium for each lecture you deliver (as much as an all-night moonlighting shift), anticipating that you will be called upon to lecture no more than one weeknight per month for 2 to 3 h each time over dinner. The company will reimburse you for travel expenses. Going forward, you may be asked to join the national speaker’s bureau, which pays more and involves delivering lectures during educational events at upscale resorts all over the world. You have extensive experience with this antihypertensive and have found that it works very well. It is reasonably priced and provides excellent results as a second-line agent.	What factors should you consider in making your decision?	*This participation will be reported to Open Payments. The impact of the reporting on one’s future career should be considered. In addition, since patients can easily search for such conflicts by clinician name, one should consider whether this might negatively impact their relationship with you. These considerations should be weighed against the opportunity for financial gain, which may be personally important, and the opportunity to participate in the advancement of medical education.*
	If you decide to accept the offer, to whom should you disclose your participation in the Speakers Bureau?	*It should be disclosed to journal editors when submitting a relevant paper, and granting agencies when submitting grants, and it may need to be disclosed to one’s institution and to medical specialty societies. In some cases, institutional rules prohibit such involvement or require a review of the contract by an institutional representative.*
Shortly after the Pfizer and Moderna mRNA vaccines against COVID-19 received emergency use authorization from the Food and Drug Administration in December 2020, you received multiple inquiries from patients—mostly older White patients—asking if they can be put on a priority list to receive the vaccine ahead of others in their age group because of their cardiac comorbidities that increase their risk of COVID-associated morbidity and mortality.	What are ethical considerations involved in this request?	*Unlike the flu vaccine, the supplies of which are typically not limited, initial COVID vaccine distribution needed to prioritize efficiency and equity. Efficiency was critical to minimize the potential of wasting vaccines (particularly while they were such a scarce resource) and to vaccinate as many people as quickly as possible to achieve herd immunity and emerge from the pandemic. Achieving this public health goal could provide potential benefit to all members of society. Just as importantly, equity was critical to ensure that vaccine allocation and distribution algorithms did not exacerbate existing disparities in COVID-associated morbidity and mortality resulting from structural racism. A system that relies on individual clinician input and advocacy to prioritize certain patients over others is likely to be both inefficient and inequitable, by adding unnecessary granularity and complexity and by favoring those who already have greater access to health care resources.*
	What are your ethical obligations as a cardiologist to your patients and to society regarding public health interventions such as vaccines?	*Many cardiac comorbidities increase the risk of COVID-associated morbidity and mortality, and the same is true for influenza. Public health measures aimed at reducing the transmission and severity of infectious disease therefore benefit many patients with cardiovascular disease. While public health authorities typically play a leading role in infectious disease mitigation, individual clinicians also play an important supportive role in encouraging patients to engage in harm-reduction strategies, as part of encouraging a healthy lifestyle. Patients with lower health literacy or limited English proficiency may be at a particular disadvantage and require additional educational time and effort. Cardiologists should also exhibit positive role modeling by getting vaccinated themselves and adhering to heart-healthy lifestyle practices.*

**Central Illustration:**
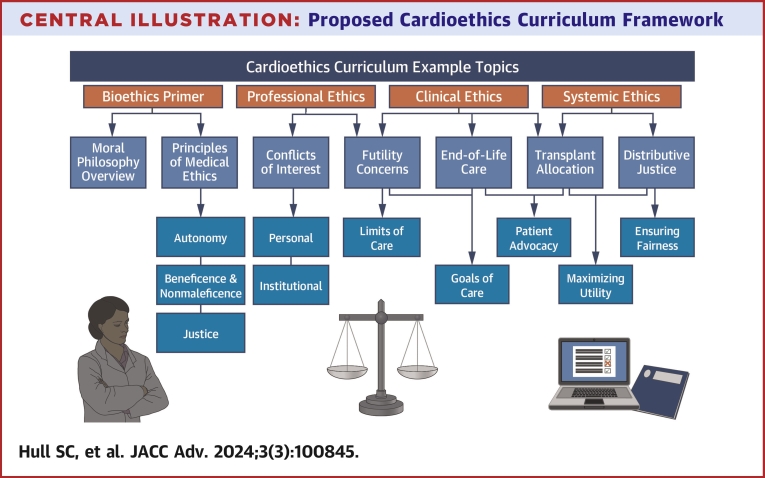
Proposed Cardioethics Curriculum Framework

## Heart transplant allocation

A physician caring for potential transplant candidates is bound by 2 sacrosanct duties that may come into conflict with each other: the duty to advocate on behalf of one’s patient and the duty to act as a steward of an incredibly scarce and precious resource.

In the United States, no resource in medicine is scarcer than transplantable organs. While the list of patients awaiting heart transplants continues to grow, the number of hearts available for transplant remained remarkably fixed at around 2,000 to 2,500 per year from 1990 to 2012, though this figure has grown to approximately 3,500 per year in 2018 to 2020 due to expanded donor criteria as well as the opioid epidemic.^4^^,^^5^ Donation after cardiac death has also contributed to increasing donor availability, though the use of normothermic regional perfusion to restore circulation in situ presents further ethical challenges in navigating how life and death are defined in light of the dead donor rule.^6^^,^^7^ Indeed, this technological advance raises the question of whether it is always accurate to conceptualize “alive” vs “dead” as a dichotomy demarcated by an instantaneous event, as there may be a liminal space in which a patient might be neither fully alive nor fully dead according to our current understanding.

Despite public health efforts to raise awareness around the importance of organ donation, there remains a wide gap between the proportion of Americans who express theoretical willingness to be an organ donor when polled vs the actual proportion registered as organ donors.^8^ Some point to long-standing mistrust of the medical establishment given its history of institutional racism,^9^ leading to a fear that they may receive less aggressive care or even be subject to a hastened death in order to procure their organs.^10^ Notably, surrogates of White brain-dead patients are significantly more likely to consent to organ donation than those of their minoritized counterparts.^11^

Most cardiologists will not play a role in transplant listing committees as part of their practices, but the vast majority will care for one or more patients being considered for heart transplant and will almost certainly participate in the care of end-stage heart failure patients during their careers. In addition to understanding reasons for organ shortages, cardiology trainees should understand the ethical issues involved in deciding who gets a heart, both in terms of listing patients for transplant on the local level, and in relation to the national system that allocates organs.

In general, we apply the principles of utilitarianism in deciding whom to list and how to allocate solid organ transplants; in other words, we attempt to derive the maximum benefit for the maximum number of individuals. As deceptively simple as this calculus seems, it is fraught with uncertainty. Exactly how do we define benefit?

One approach that seems intuitive is to allocate organs to the very sickest individuals who have the greatest need and therefore stand to benefit the most. However, this approach can present a significant challenge in that there is no clear dividing line between patients who desperately need an urgent transplant and patients who are so sick that they may not be able to derive meaningful benefit from a transplant. For instance, a patient in cardiogenic shock may develop irreversible multi-organ failure that might be refractory to even the most intensive support measures. In some cases, we can offer rescue therapy such as temporary or durable mechanical support, but these are fraught with their own risks. How sick is too sick for a patient to remain a transplant candidate?

Should nonmedical issues such as putative contributions to society or ability to pay play a role in organ allocation? This approach on its face seems unfair. For example, when Steve Jobs (a California resident) received a liver transplant in Tennessee, media reports arose pointing out the unfairness of increased access among those with substantial resources.^12^ Jobs was able to receive a transplant more quickly than most because he had the financial resources to put himself on multiple waiting lists throughout the United States. Might one argue that Jobs, through his innovations at Apple, revolutionized digital life in this country and around the world and therefore had a life more worthy of saving? Arguments about worthiness predicated on “service to society” featured prominently in the deliberations of the Seattle “God Committee” tasked with deciding which patients would have access to lifesaving but extremely scarce kidney dialysis machines soon after this technology was invented in the early 1960s.^13^ Of course, this took place a decade before the field of bioethics emerged, and indeed helped to spur its development. In the modern era, these sorts of subjective value judgments are generally rejected by the medical profession due to their inherent bias. At least in theory, we now putatively strive to treat all lives as equally valuable. Yet in the United States, we still practice medicine in a system where access to care is largely dictated by insurance coverage and ability to pay either through this insurance or directly out of pocket. Those who cannot pay simply do not have access, or have reduced access, to high-quality medical care, and often their only option is temporizing measures in the emergency department to which they are legally entitled regardless of ability to pay.^14^

To what extent should psychosocial criteria influence listing? This seems reasonable to a certain extent given that patients receiving a solid organ transplant are expected to be good stewards of the precious and scarce resource bestowed upon them. We generally do not hesitate to perform percutaneous coronary intervention on a patient who smokes with no intentions of quitting, but we expect a patient with end-stage heart failure to demonstrate sustained abstinence to be eligible for transplant due to scarcity of the resource and published data on worse post-transplant outcomes for those who smoke.^15^ However, some would argue that this approach may lead to the slippery slope of moral judgment. Convicted felons have demonstrated a clear inability to comply with instructions (in their case with the laws of society) so does that mean they should not receive a transplant under any circumstances? If so, is it because they are less likely to have access to care or to be adherent to medical therapy, or because they are less “deserving” of such a scarce resource because they have already placed a burden on society? What about otherwise law-abiding citizens with multiple speeding tickets? Surely their risks of death from motor vehicle collisions are higher, but is that sufficient to justify their being excluded as potential transplant recipients? Where and how do we draw the line? While it is important to maximize the potential benefits of scarce resources, there is broad ethical consensus that we should strive to treat all lives as equally valuable and refrain from using moral desert as a factor in resource allocation. Furthermore, strict psychosocial criteria, such as requiring a dedicated caregiver after transplant, may have the untended consequence of exacerbating pre-existing inequities that already disproportionately affect vulnerable populations.

Regardless of how we decide to list patients and allocate organs, there often remains a concern that physicians will try somehow to manipulate the rules in order to maximize the chances that their patients will receive a transplant. Indeed, an analysis by Parker et al^16^ expresses concern that the allocation algorithms may promote “gaming the system” by heart transplant programs and physicians who may alter their patients’ inotropic or mechanical support therapy to move them higher on the list. Specifically, treatment of listed patients who are not in shock with inotropes and temporary mechanical support is not uncommon, and regional differences in overtreatment appear to correlate with competition for donor hearts.^17^

We would argue that the majority of transplant physicians, like the majority of physicians in general, entered the profession because they truly want to make people better and to do the right thing. The right thing, unfortunately, is not always clear when the duty of advocating for an individual patient conflicts with the duty of resource stewardship. Who could fault a physician who, if choosing between 2 roughly equivalent therapeutic options, would pick the option that would increase her patient’s chance of receiving a transplant in a timely fashion? An ideal allocation framework would prioritize objectivity (criteria that are less easily altered by clinical decision-making), transparency (criteria that are clearly delineated rather than debated on a case-by-case basis, a posteriori), and consistency across transplant centers so we do not send patients mixed messages or unfairly favor those who already enjoy significant financial privilege. The recent changes to heart transplant allocation criteria have attempted to balance these concerns with optimization of outcomes.^18^, ^19^, ^20^ However, as noted above, these changes may nonetheless lead to greater use of temporary mechanical circulatory support as has already been observed.^21^

As for patients who are not eligible for cardiac transplant, many receive costly devices such as left ventricular assist devices (LVADs), biventricular pacemakers, and implantable cardioverter-defibrillators to enhance longevity and quality of life. While implantable devices are hardly as scarce as organ transplants, their high cost and potential risks mean we should not offer them indiscriminately to patients with heart failure who may qualify based on narrow physiologic criteria but may not stand to benefit within a larger biopsychosocial context. Performing profitable procedures that ultimately fail to meaningfully lengthen or improve quality of life—and as such might be described as futile—does a disservice to patients and undermines physician trustworthiness and accountability.

## Futility

How should cardiologists approach the evaluation of potential futility? First, it is important to note that the concept of futility can be fraught with value judgments, as whether an intervention is truly futile often depends on the goals that one is trying to achieve with it. An implantable cardioverter-defibrillator is an effective intervention to prevent sudden cardiac death due to ventricular arrhythmia in patients who meet guideline criteria, but one that could be considered “futile” to an advanced heart failure patient whose goals are strictly limited to interventions that improve symptoms or quality of life. As such, some ethicists argue that the term “futile” should be avoided in most cases.^22^ At the very least, it is helpful to break this broad concept down into 4 categories: physiologic, quantitative, qualitative, and psychosocial.

Physiologic futility, the clearest and least controversial, refers to treatments or interventions that cannot be expected to provide any benefits because they have no chance of succeeding from an anatomical, physiological, or biochemical standpoint. Simple examples include prescribing antianginal therapy for chest pain due to costochondritis or shocking true asystole.

Quantitative futility similarly encompasses interventions whose potential for benefit approaches zero (alternatively expressed as <1% or similarly small chance of success). For example, cardiologists may face the example of a critically ill, septic patient in cardiogenic shock with severe aortic regurgitation whose multiple comorbidities preclude transplant or LVAD and who is actively dying despite the infusion of multiple pressors and mechanical ventilation with 100% oxygen. If this patient arrests with pulseless electrical activity, one cannot reasonably expect any benefit to be derived from chest compressions, though temporary restoration of a (minimally) perfusing rhythm is theoretically possible.

Qualitative futility encompasses interventions that may be physiologically sound with respect to an isolated disease state but that may not provide meaningful benefit in the broader context of overall health. Importantly, this often depends on a patient’s goals. While someone with unstable angina, reduced ejection fraction, and multivessel coronary disease would generally benefit from coronary artery bypass grafting, this might make less sense in someone with advanced metastatic cancer who is not expected to live more than a few months. Given the inherent subjectivity involved, such a decision generally requires an open and honest discussion between patients and clinicians, considering medical facts as well as patient goals and values in order to make a shared decision.

Psychosocial futility is the most challenging for clinicians and can be readily misapplied. It refers to treatments that are medically appropriate but that may not deliver a sustained benefit to a patient who engages in behaviors that diminish the efficacy of the intervention in question. Patients with angina who are unwilling to take any medications even for a short time would be poor candidates for elective percutaneous coronary intervention with a stent, as the risk of stent thrombosis in this case could leave them in worse condition than if they were never to receive the intervention. An often contentious scenario involves the question of whether to offer serial valve replacements to a patient with recurrent injection drug use-associated endocarditis.^23^ While these patients are engaging in self-destructive behavior, addiction is now understood to be a medical illness for which evidence-based treatments exist.^24^ The underlying disease of addiction should be treated to the greatest extent possible before one can fairly say it is futile to treat a cardiac manifestation of this disease. Furthermore, invoking psychosocial futility has the potential to exacerbate systemic inequities by making assumptions about patients’ ability to adhere to medical regimens.

## Withdrawing life-sustaining treatments

Implantable life-sustaining devices such as defibrillators and LVADs make the issue of life-sustaining treatment withdrawal particularly salient in cardiology, as these may symbolically become part of a patient’s body in a way that external supportive measures such as hemodialysis and mechanical ventilation do not. There is broad consensus in the medical profession that withdrawing life-sustaining measures is morally permissible provided this is consistent with patient wishes.^25^ As such, most clinicians feel comfortable with terminal extubation after reaching a shared decision to focus on comfort measures only. However, making a similar decision with respect to device inactivation may be more emotionally challenging because devices cannot be neatly separated from patients in the way a ventilator can, a distinction that has been described as the “biofixtures” argument.^26^

Challenges regarding device inactivation can be exacerbated by the lack of understanding important nuances. If a patient wishes to receive only comfort measures and has a pacemaker-defibrillator, the defibrillator should be deactivated to prevent shocks that may occur in the process of dying, as these would only needlessly and painfully prolong it. There is no need to deactivate a pacemaker in this case, as pacing is usually imperceptible and capture is eventually lost in terminal metabolic dysregulation. In most cases, pacing causes neither pain nor prolongation of dying and as such does not interfere with comfort measures as a defibrillator can.^27^

Of course, terminally ill patients may request pacemaker deactivation if they are intractably suffering and wish to minimize any life-prolonging measures. Clinicians may be particularly uncomfortable with a request to deactivate a pacemaker in a chronotropically incompetent patient who is unable to sustain adequate cardiac output without pacemaker support. While this may feel like hastening death, there is ethical consensus that this is morally equivalent to terminally extubating a patient with ventilator-dependent respiratory failure. In both cases, the clinician is not actively hastening death but rather removing artificial life-sustaining measures and passively allowing the underlying terminal illness to take its course. Still, some clinicians may express understandable reticence to deactivate a constitutively active device if doing so would immediately precipitate a patient’s certain death. The Heart Rhythm Society published a consensus statement in 2010 that provides a thoughtful analysis of this decision-making process.^25^

The deactivation of LVADs is thornier still. Ethicists generally agree that withdrawing an intervention is morally equivalent to withholding an intervention,^25^ because withdrawing most interventions leaves the patient in no worse condition than if they had never received them in the first place and simply allows the underlying disease process to run its course. However, deactivating LVADs may lead to pump thrombosis or aortic regurgitation that patients would not have had if they had never received an LVAD. This is not to argue that it is never ethical to deactivate an LVAD, but clinicians and patients may express legitimate reluctance to do so even if they are comfortable withdrawing other support measures. Accordingly, judicious advanced care planning is of particular importance prior to LVAD implantation, especially when undergone for destination therapy when patients are not candidates for transplant. Cardiologists must understand these ethical distinctions in order to explain to patients and assist them in advance care planning that accurately reflects patient values and preferences.

## Advance care planning and care at the end of life

Because cardiologists manage a range of acutely and chronically life-threatening conditions, it is critical that cardiology trainees become adept at navigating end-of-life care with competence and compassion.

If patients with advanced chronic illness can articulate their goals and values, it is helpful to elicit these through thoughtful discussion before they progress to the end stages of their diseases. This includes a clear description of what advanced care directives such as “do not resuscitate” or DNR actually entail. Some patients who initially state they “want to be DNR” if questioned more deeply might clarify that they would indeed want cardiopulmonary resuscitation in the setting of a reversible cause with a meaningful chance of recovery. For example, an older individual with otherwise well-compensated congestive heart failure might desire intubation for acute pneumonia if necessary until respiratory status improves after a few days of antibiotics and diuretics. Ventricular tachycardia is often immediately reversible with defibrillation and may not even require chest compressions or intubation if a patient immediately regains consciousness. What patients may mean when they request DNR status is that they do not want to be maintained for a protracted time on invasive cardiopulmonary support with little chance of meaningful functional recovery afterward. Alternatively, a patient with significant cardiac disease may indeed want to forego all resuscitative interventions in the setting of developing progressive dementia, advanced stage cancer, refractory cardiac symptoms, or simply after having lived a long life. Such a patient may be choosing a relatively quick and painless death over prolonged suffering.

Avoiding medical jargon and clarifying patient wishes in lay terms is critical in making this distinction and maximizing patient autonomy. Encouraging patients to create a living will (a document outlining their wishes in different end-of-life scenarios) or to appoint a surrogate decision-maker (or medical power of attorney) may be helpful, though these legal measures may not be necessary if patients have clear and open discussions with their surrogates, families, and clinicians who should document their wishes in patient medical records. Persistent misconceptions and misinterpretation of prior documentation can significantly limit the utility of advance care planning in practice, as it may be difficult to anticipate the many complexities of a specific clinical context. As such, advance care planning does not obviate the need for clinicians to engage in earnest and empathic shared decision-making discussions with patients and surrogates in real time.^28^ Indeed, while the 2022 AHA/ACC/AFSA (American Heart Association/American College of Cardiology/American Heart Failure Society of America) guidelines for the management of heart failure suggest advance care directives as a Class 2a recommendation, they make a Class 1 recommendation that “palliative and supportive care—including high-quality communications, conveyance of prognosis, clarifying goals of care, shared decision-making, symptom management, and caregiver support—should be provided to improve quality of life and relieve suffering.”^29^

When caring for critically ill patients who cannot express their wishes, surrogate decision-makers can help guide decision-making using the “substituted judgment” standard to intuit and articulate what patients would want in such circumstances based on prior knowledge of patient goals and values. When patient preferences remain unclear, clinicians often apply the “best interest” standard to these discussions, offering suggestions based on what they perceive a reasonable person might decide while recognizing that there may not be a clear best option.^30^ Although it is critical not to offer false hope of unrealistic recovery, it is equally important not to push surrogates toward comfort-only measures before they have had time to process a dire prognosis. Overly aggressive attempts to limit care to palliative measures before surrogates are ready can often backfire and make them feel fearful or distrustful, which in turn may lead them to insist upon overly aggressive care that is unlikely to provide meaningful benefit. Similarly, approaching these conversations with cultural humility is critical to respecting patients and surrogates who may have religious or cultural values that differ from those of clinicians.

## Conflicts of interest regarding referrals and appropriate use

Discussion of the business aspects of medicine is often lacking during training, so fellows and other trainees may not give much thought to reconciling medical ethics with business ethics before entering practice. Still, many have seen patients expecting a yearly “screening” stress test because their prior cardiologist insisted upon it, despite the absence of evidence supporting this practice. This is particularly problematic when the same cardiologist who orders the test is the one who will be reimbursed for performing the test, as it represents a potential financial conflict of interest. On the other hand, if stress testing is ordered for an appropriate indication by a cardiologist who also runs the hospital’s stress lab, is this really a conflict of interest or just good evidence-based medical care?

Furthermore, inappropriate stress tests may be ordered to evaluate chest pain that is almost certainly noncardiac based on history and presentation, either because patients insist or because physicians fear medical-legal repercussions. Guidelines-driven practice and adherence to appropriateness criteria in stress testing can provide a helpful counterbalance.^31^ However, it is important for cardiovascular professionals to understand how to counsel patients requesting inappropriate testing, as it is not benign. Incidental or inconclusive findings can prompt invasive testing that can lead to avoidable complications or unnecessary anxiety. This is also a wasteful and inappropriate allocation of resources, of which we should be especially mindful in the United States given that we spend roughly twice per capita on health care expenditures compared with other wealthy nations.^32^ The 29th Bethesda Conference in 1997 provided a comprehensive framework for approaching ethical issues in managed care.^33^ Regarding the practice of defensive medicine, while medical malpractice reform is surely needed, trainees should know that the only practices demonstrated to be protective from a medical-legal standpoint are effective communication with patients and clear documentation in the medical record.^34^ Inappropriate testing does not prevent litigation, and it conflicts with both the duty to avoid patient harm and the duty of resource stewardship.

## Distributive justice and fairness

The duty of the medical profession to serve as judicious stewards of societal resources must extend beyond allocation of solid organ transplants and avoidance of unnecessary procedures. The COVID-19 pandemic highlighted painful lessons regarding critical care resource allocation and institutional racism. Hospitals in hardest hit areas found themselves forced to ration critical care resources such as ventilators, extracorporeal membrane oxygenation circuits, and intensive care unit beds when demand exceeded supply. And while machines can be manufactured and nonclinical spaces can be repurposed in a relatively short period of time, finding enough highly skilled staff such as intensivists, critical care nurses, and respiratory therapists can prove even more challenging as these skills require years of training. Even institutions not overwhelmed to the point of instituting crisis standards of care and triage protocols adopted contingency standards of care to conserve resources.^35^ Elective cardiac imaging and procedures were often deferred in the first wave of the pandemic, and some cardiology staff were “redeployed” to intensive care unit settings to help care for critically ill COVID patients. Outpatient cardiology visits were largely converted to telehealth visits to further conserve personal protective equipment and minimize exposures for staff and patients alike.

Whenever shifts in resource allocation on this scale occur, medical institutions must be mindful to ensure fairness and equity. Minoritized populations already have reduced access to health care and poorer health outcomes in the United States, a pattern that has been starkly reflected in morbidity and mortality trends during the COVID-19 pandemic.^36^ Without intentional safeguards, implementing contingency standards of care risks exacerbating these disparities. For example, while telehealth may improve health care access for many, less privileged individuals are more likely to have limited connectivity or limited technical literacy and thereby risk becoming “doubly disadvantaged” if they are unable to use digital telehealth platforms as effectively as their more socioeconomically privileged counterparts.^37^

Cardiovascular disease, like COVID, disproportionately affects minoritized populations due to social determinants of health, such as limited access to education and nutritious food.^38^^,^^39^ Overcoming structural disparities and institutional racism requires a shift in focus from equality (treating all patients the same way in theory—though we often fall short in practice) to equity (treating patients differently in a manner commensurate with their individual needs). For example, patients with limited health literacy may require more frequent counseling and more extensive ancillary support than their more privileged counterparts to promote better adherence to complex medical regimens or heart-healthy lifestyles.^40^ On a societal level, clinicians—and especially cardiologists—have an obligation to advocate for policy changes such that all members of society have adequate access to health education and nutritious food from an early age.

## Conclusions

While this is hardly an exhaustive discussion of ethical issues cardiologists can expect to encounter in clinical practice, we chose to focus on this somewhat limited scope to illustrate examples of particular relevance to clinicians caring for patients with cardiovascular disease. In addition to this narrative outline, we have included a set of cases and questions (Table 3) to frame small group discussion sessions and lower the “activation energy” needed to institute cardioethics education by making this content more pedagogically accessible. We have also included primers on moral philosophy (Table 1) and bioethics principles (Table 2) to frame ethical discourse and promote practical moral reasoning for those without formal ethics training. We hope this proposed “cardioethics” curriculum can serve as a launching pad for more formal and comprehensive approaches to ethics education in cardiology fellowship, in addition to continuing medical education for all cardiovascular professionals. Cardiology trainees and practitioners should be encouraged to reflect upon and debate these and other difficult questions, carefully considering their duties to individuals as well as their duties to society. Complex ethical decision-making in cardiology often requires a combination of extensive technical knowledge, nuanced appreciation of individual patient goals and values, and thoughtful application of ethical principles. Cardiologists have an exceptionally broad toolkit of highly technical and complex interventions to address high-stakes disease states. We would be well served to maintain a similarly broad ethical toolkit to provide the best and most comprehensive care for our patients.

## Funding support and author disclosures

The authors have reported that they have no relationships relevant to the contents of this paper to disclose.
